# Combined Anterior Sclera Staphylectomy and Vitrectomy with Anterior Sclera Staphyloma and Vitreous Hemorrhage Occurring 38 Years after Cataract Surgery

**DOI:** 10.1155/2011/340859

**Published:** 2012-01-23

**Authors:** Qinxiang Zheng, Ronghan Wu, Wensheng Li

**Affiliations:** Eye Hospital, Wenzhou Medical College, 270 Xueyuan Road, Zhejiang, Wenzhou 325027, China

## Abstract

*Introduction*. To report a case of anterior sclera staphyloma and vitreous hemorrhage occurring over 38 years after bilateral cataract surgery. *Methods*. A 58-year-old man presented with anterior sclera staphyloma and vitreous hemorrhage in the right eye, after bilateral cataract surgery, over 38 years ago. We performed combined anterior sclera staphylectomy and vitrectomy of right eye for anterior sclera staphyloma and vitreous hemorrhage. *Results*. Forty-eight months after the combined surgery, best-corrected visual acuity was 0.3 (+10.00/−4.50 × 60) with eutopic stitches of the corneoscleral junction on the superior nasal quadrant and a stable ocular surface. *Conclusions*. This is the first reported case of anterior sclera staphyloma with vitreous hemorrhage successfully managed by combined surgery.

## 1. Introduction

Common reasons for staphyloma formation include surgery, trauma, inflammation, glaucoma, high myopia, malnutrition, and developmental abnormalities [1, 2]. Clinically, staphyloma is an uncommon complication due to various causes, whereas vitreous hemorrhage may be encountered more frequently. However, there has been no report in the literature of anterior staphyloma complicated with simultaneous vitreous hemorrhage. We report an unusual case of anterior sclera staphyloma involving vitreous hemorrhage with descriptions and insights that may help surgeons to further manage this special situation.

## 2. Case Presentation

**Figure 1:**
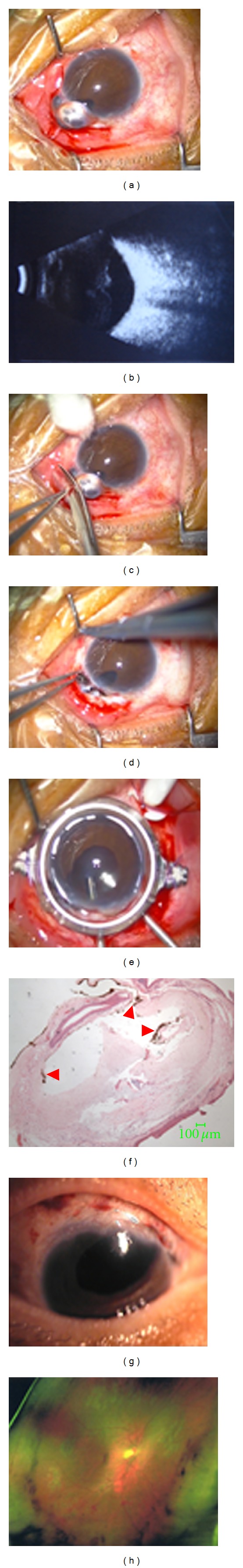
Clinical photograph, ultrasonographic fundus photograph and histopathology of right eye. (a) A brown protruding bulge occupied the entire nasal quadrant with a clear boundary seen at the outer edge of the corneoscleral limbus. The cornea was transparent, the anterior chamber was shallow and about 1/3CK, the pupil shifted up and dwindled with an apparent discoria. (b) B-scan showed vitreous opacity with posterior vitreous detachment. (c) The bulbar conjunctiva around the anterior staphyloma on the superior nasal quadrant was separated; the prolapsed uveal tissues and vitreous body were pruned during operation. (d) After surgical removal of the anterior staphyloma, the apparent thinning and perforated sclera at 12:00–2:30 position and iridoleptynsis on the superior nasal quadrant was observed. (e) The shifted-up iris was cut to recover a circular pupil and to clear vitreous hemorrhage by vitrectomy. (f) The removed specimens were confirmed as anterior staphyloma with the existence of pigment cells (arrowhead) by pathological examination. (g, h) Re-examination three months after operation: the right cornea was transparent, the stitches of corneoscleral junction on the superior nasal quadrant were eutopic, the anterior chamber was deep and the pupil was circular; the retina was procumbent by Panoramic200.

On July 15, 2005, a 58-year-old man presented with sudden onset of severe pain and blurred vision in his right eye, without significant irritation, of one day's duration. The eye pain was alleviated after half an hour; however, visual acuity was significantly decreased. On examination, visual acuity in the right eye was HM/60 cm with accurate light projection in every direction; best-corrected visual acuity in the left eye was 0.6 (+10.00/−3.50 × 45). Intraocular pressure (IOP) was 7.8 mmHg in the right eye and 15.0 mmHg in the left eye. Examination of the right eye showed conjunctival congestion, a brown protruding bulge occupying the entire nasal quadrant with a clear boundary seen at the outer edge of the corneoscleral limbus. The cornea was transparent, the anterior chamber was shallow and about 1/3CK, the pupil shifted up and dwindled with an apparent discoria, the light reaction disappeared, and the fundus was not clear (Figure 1(a)). The right eye vitreous had an obvious opacity with posterior vitreous detachment (PVD) (Figure 1(b)) by B-scan. Examination of the left eye showed a pupil shifted up in a lip-like shape, with a defect at the top of the iris and a flocculent vitreous opacity. The boundary of the optic disc was clear. There was mild sclerosis of the fundus artery and no macular reflex, and the retina was procumbent. In 1967, the patient had undergone cataract surgery in a local hospital due to congenital cataracts in both eyes. He has been wearing spectacles as treatment after the operation without discomfort. There was no past history of trauma in either eye. Apart from a 10-year history of hypertension, he was otherwise fit. A diagnosis was made of anterior sclera staphyloma and vitreous hemorrhage in the right eye.

On July 19, 2005, the patient underwent anterior scleral staphyloma resection and repair with sutures placed, plus vitrectomy, coreoplasty, and endophotocoagulation under general anesthesia. The bulbar conjunctiva around the anterior staphyloma on the superior nasal quadrant was separated; the prolapsed uveal tissues and vitreous body were pruned during operation (Figure 1(c)). We observed an apparent thinning and perforated sclera at the 12:00–2:30 position and iridoleptynsis on the superior nasal quadrant. Subsequently, we placed an apposition suture and covered the wound using the anadesma and conjunctiva (Figure 1(d)). We performed a vitrectomy with three incisions at the 7:00, 10:00, and 11:00 o'clock positions (normally at 8:00, 10:00, and 2:00 o'clock positions) of 3.5 mm behind the corneoscleral limbus and cut the portion of the shifted-up iris. We noticed a circular pupil, the hemorrhagic vitreous opacity, and the discus opticus with normal size and shape (Figure 1(e)). In addition to the inferior nasal quadrant, scattered patchy hemorrhage points were seen in the remaining parts of the retina. The patient was then subjected to laser photocoagulation followed by local conventional anti-inflammatory and mydriasis treatment after the operation. The removed specimens were confirmed as anterior staphyloma on histological examination (Figure 1(f)). Reexamination three months after surgery showed a corrected visual acuity of 0.3 (+10.00/−4.50 × 60) and IOP of 14.0 mmHg in the right eye. The right cornea was transparent, the stitches at the corneoscleral junction on the superior nasal quadrant were eutopic, the anterior chamber was deep, and the pupil was circular (Figure 1(g)); the light reflex was slow, and the retina was procumbent by Panoramic 200 (Figure 1(h)). The patient's last visit was four years after surgery with the clinical findings identical to those at three months after surgery.

## 3. Discussion

The anterior staphyloma of this patient was caused by previous surgery. In 1967, due to the limitations of surgery at that time and because postoperatively the iris was missing at the top of both eyes, we speculated that the sutured incision was malaligned and the top of the iris prolapsed, formed an incarceration. Although the intraocular pressure was low, the tissues including the sclera, choroid, or the iris protruded, expanding outward and finally forming a scleral staphyloma that resembled a purple black grape-shaped bulge, all secondary to postoperative damage to the eyeball wall that led to reduced resistance. This may be the main reason for the formation of the anterior scleral staphyloma in this patient. The case we report has similar characteristics to that described by Buckley and associates [3] that showed formation of a ciliary staphyloma induced by a corneoscleral tunnel incision cracking following intracapsular cataract surgery. The cause of vitreous hemorrhage was the deep retinal hemorrhage caused by rupture of the deep retinal capillary plexus, located between the outer plexiform layer and the inner nuclear layer, as commonly found in fundus bleeding caused by vascular lesions in patients with diabetes and high blood pressure. Since this patient had no history of diabetes, we largely concluded that the fundus hemorrhage was caused by hypertension.

The biggest challenge presented to us in this case was how to manage the simultaneous anterior staphyloma and vitreous hemorrhage. As a result of the large anterior staphyloma and broad scope of resection, and for the sake of better restoring the local anatomy and visual acuity after surgery, we repeatedly recommended the use of an allogeneic scleral patch or repair of the dura mater in this patient [4]. However, the patient firmly refused for religious reasons. Thus, we could only employ an anterior staphyloma resection, suturing, and repair, plus vitrectomy, coreoplasty, and endophotocoagulation. In this operation, we resected the anterior staphyloma, repaired the ruptured sclera, and implemented an apposition suture. Cracking and other complications did not occur during the follow-up period. Since the patient refused allograft scleral transplantation, the only shortcoming was greater postoperative astigmatism.

In summary, this patient presents a very rare and complex case showing simultaneous occurrence of an anterior staphyloma and vitreous hemorrhage that occurred 38 years after cataract surgery. The reasons underlying both pathological changes are unique. We resolved the coexistence of an anterior staphyloma and vitreous hemorrhage with a one-time remedy by employing an anterior staphyloma resection, and suture and repair operation, plus vitrectomy, coreoplasty, and endophotocoagulation. After four years of followup, except for the larger postoperative astigmatism, no other complications occurred. Not only did the eyeball survive, but good vision was also restored to the patient.
